# Triglyceride/high-density lipoprotein ratio as a predictor for insulin resistance in a sample of healthy Iraqi adults

**DOI:** 10.25122/jml-2022-0239

**Published:** 2023-05

**Authors:** Zahraa Abdul Ghani, Hussein Qaddori, Qasim Al-Mayah

**Affiliations:** 1. College of Pharmacy, AL Bayan University, Baghdad, Iraq; 2. Department of Physiology, College of Medicine, Al Nahrain University, Baghdad, Iraq

**Keywords:** insulin resistance, density lipoprotein ratio, triglyceride/high-density lipoprotein ratio

## Abstract

Insulin resistance (IR) is a common pathophysiological condition associated with many metabolic diseases, including obesity, prediabetes, type 2 diabetes mellitus (T2DM), and cardiovascular disease. The widely used homeostasis model assessment for IR (HOMA-IR) is usually used to estimate IR. However, this model cannot be used for screening IR due to several logistic difficulties, such as costs and insulin instability, which are essential for measurement. Thus, finding feasible alternatives is of paramount importance. This study aimed to evaluate the value of triglyceride/high-density lipoprotein-cholesterol (TG/HDL-c) ratio in predicting IR in healthy adult individuals. The study involved 83 euglycemic non-diabetic adults (≥45 years old). Lipid profile, fasting insulin, fasting blood sugar (FBS), and glycated hemoglobin were measured for all participants. The TG/HDL-c ratio was calculated by dividing TG by HDL. Insulin resistance was calculated using the HOMA-IR formula. The receiver operating characteristic (ROC) curve was used to evaluate the predictive value of the TG/HDL-c ratio. The prevalence of IR among healthy adult Iraqis was 28.92%. Subjects in the IR group showed a higher TG/HDL ratio than the insulin-sensitive group (3.69±0.68 versus 2.71±1.0) with a significant difference. The area under the curve (AUC) for this ratio was 0.849, 95% CI= 0.763-0.935, p<0.002. The sensitivity and specificity of the test were 83% and 81%, respectively, at a best cut-off value of 3.1 (TG/HDL ratio). The prevalence of IR among healthy adult Iraqis was 28.92%. Triglyceride/HDL-c ratio had a very good predictive value for IR.

## INTRODUCTION

Insulin resistance (IR) is a concerning indicator for the development of serious pathological conditions such as type 2 diabetes mellitus (T2DM), metabolic syndrome, atherosclerosis, and hypertension. Thus, regular screening for IR in healthy individuals could be a good strategy to reduce the incidence of these diseases. The current standard test for measuring IR is the homeostasis model assessment for IR (HOMA-IR), but it is often impractical in clinical settings primarily due to differences in gender, race, age, lifestyle (exercise), dietary habits, and metabolic assets. [1]. Therefore, it is essential to identify a practical and feasible alternative for the early detection of IR.

Dyslipidemia has been suggested as a potential tumor biomarker, with the triglyceride to HDL cholesterol (TG/HDL) ratio being associated with various cancers [2]. This ratio is thought to reflect insulin resistance (IR), a key factor in many metabolic diseases, including cancer [3]. High-density lipoprotein cholesterol (HDL-C) levels and hypertriglyceridemia are known to be key factors in the pathophysiology of IR [4,5]. On the other hand, fatty acid production is increased in cases of IR. This leads to increased triglycerides (TG) as well as very low-density lipoprotein (vLDL) production [6]. Accordingly, it is reasonable that IR is associated with high levels of TG and low levels of HDL. This, in turn, reduces HDL-C levels in individuals with IR. Consequently, TG/HDL-C ratio has been proposed as a surrogate for HOMA-IR to predict IR. While several studies worldwide have shown the feasibility of using the TG/HDL-C ratio to predict IR, variations in TG and HDL-C levels among different racial and ethnic groups have been observed, which may affect the accuracy of the ratio as a predictor [7,8]. In this study, we aimed to investigate the predictive value of the TG/HDL-C ratio in predicting IR in a sample of apparently healthy individuals from Iraq.

## MATERIAL AND METHODS

The study enrolled 85 apparently healthy adult subjects of both sexes from Al Bayan University and Al-Mustafa University College, Baghdad, Iraq, between September 2021 and April 2022. Participants were euglycemic non-diabetic adults ≥45 with HbA1C less than or equal to 6.5%. Age, gender, smoking status, place of residence, and family history of diabetes data were collected. The participant's height (m), body weight (kg), BMI, and waist circumference (WC) were measured.

### Collection and preparation of samples

Participants in the study were instructed to fast for 8 hours prior to blood sample collection. The following morning, 5 ml of venous blood was collected from each participant. The blood sample was split into two aliquots of 3 ml and 2 ml. Three milliliters of blood were placed in a plain tube. The serum was recovered by centrifugation. Two milliliters of blood were placed in ethylenediaminetetraacetic acid (EDTA) tubes for hematological tests. Fasting blood glucose, glycated hemoglobin (HbA1c), and lipid profiles were measured. Insulin resistance was calculated using the HOMA-IR formula as follows:

$$\begin{array}{c} \text{HOMA-IR}=(\text{FBS }(\text{mg}/\text{dl})\;\times \text{ fasting insulin }(\text{mU}/\text{L})/\text{405}\\ \text{The TG}/\text{HDL-c ratio was calculated by dividing TG}\\ \text{concentration by HDL concentration.} \end{array}$$

### Statistical analysis

The statistical analysis was performed using SPSS software version 25.0 (SPSS, Chicago). Continuous data were presented as mean and standard deviation and analyzed using a Student t-test, while categorical variables were presented as percentages and integers and analyzed using the Chi-square test. To determine the diagnostic value of the TG/HDL-c ratio in predicting IR, receiver operating characteristic (ROC) curve analysis was performed under the non-parametric assumption, and the cut-off value with the highest sensitivity and specificity was calculated using the Youden Index [9]. A p-value of less than 0.05 was considered statistically significant.

## RESULTS

### Demographic and clinical characteristics

The mean age of the participants was 54.17±9.24 years, with a range of 45-80 years, and 63.86% were males. Smoking and family history were relatively common, accounting for 39.76% and 53% of the subjects, respectively. The mean weight and height of the subjects were 82.73±11.94 kg and 166.67±5.83 cm, respectively, resulting in a mean BMI of 29.65±3.70 kg/m2. Finally, the mean waist and hip circumferences were 93.42±19.2 cm and 105.75±21.62 cm, respectively (Table 1).

**Table 1.** Baseline characteristics (N=85)

Variables		Value
**Age, years**	Mean±SD	54.17±9.24
Range	45-80
**Gender**	Male	53(63.86%)
Female	30(36.14%)
**Smoking**	Never	50(60.24%)
Ex/current	33(39.76%)
**Family history**	No	39(47%)
Yes	44(53%)
**Weight, kg**	Mean±SD	82.73±11.94
Range	58-112
**Height, cm**	Mean±SD	166.67±5.83
Range	151-179
**Waist circumference**	Mean±SD	93.42±19.2
Range	59-144
**Hip circumference**	Mean±SD	105.75±21.62
Range	64-152
**BMI, kg/m^2^**	Mean±SD	29.65±3.70
Range	22.2-36.96

Most participants had a normal lipid profile, with only a small minority exceeding normal limits. The mean serum levels of total cholesterol (TC) and triglycerides (TG) were 182.78±26.96 mg/dl and 146.65±49.79 mg/dl, respectively. High-density lipoprotein (HDL) levels ranged from 41 to 59 mg/dl with a mean of 49.03±4.64 mg/dl. The mean for low-density lipoprotein (LDL) and very low-density lipoprotein (VLDL) were 99.44±19.28 mg/dl and 34.28±9.16 mg/dl, respectively, and were almost within the normal range. The calculated TG/HDL ratio was 3.0±1.07, as shown in Table 2.

**Table 2.** Lipid profile of participants

Variables		Value
**Total cholesterol, mg/dl**	Mean±SD	182.78±26.96
Range	133-241
**Triglycerides, mg/dl**	Mean±SD	146.65±49.79
Range	81-328
**HDL, mg/dl**	Mean±SD	49.03±4.64
Range	41-59
**LDL, mg/dl**	Mean±SD	99.44±19.28
Range	67.8-145
**VLDL**	Mean±SD	34.28±9.16
Range	16.2-56
**TG/HDL**	Mean±SD	3.0±1.07
Range	1.5-7.29

The mean fasting blood sugar and HbA1c were normal, with values of 89.45±8.0 mg/dl and 5.07±0.54%, respectively. The mean fasting insulin level was almost normal, ranging from 3.62-25.3 mIU/L. However, the calculated HOMA-IR showed considerable variation, with a mean of 2.37±1.18 and a range of 0.46-5.37, as shown in Table 3.

**Table 3.** Insulin resistance-related parameters

Variables		Value
**FBS, mg/dl**	Mean±SD	89.45±8.0
Range	72-109
**HbA1c, %**	Male	5.07±0.54
Female	4.19-6.3
**Fasting insulin, mIU/L**	Never	10.9±5.15
Ex/current	3.62-25.3
**HOMA-IR**	No	2.37±1.18
Yes	0.46-5.37

### Insulin resistance rate

**Figure 1.**
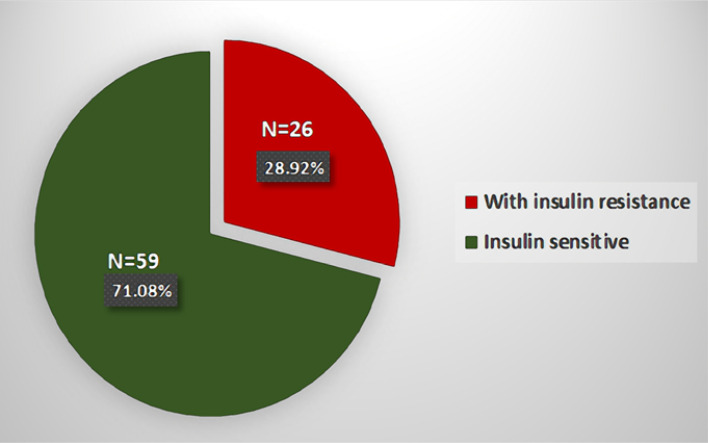
IR rate in healthy subjects

Out of 85 apparently healthy subjects, 26 subjects (28.92%) had IR, while the other 59 subjects (71.08%) were insulin-sensitive (Figure 1).

### The association between demographic and clinical characteristics and IR

The mean body weight and BMI were significantly higher in subjects with IR than in those with insulin sensitivity, with values of 87.21±12.12 kg and 31.37±3.84 kg/m2, respectively, compared to 80.92±11.84 kg and 28.95±3.45 kg/m2, respectively (Table 4). Although there was a higher proportion of females in the IR group than in the insulin-sensitive group (50% vs. 30.51%), the difference was not statistically significant.

**Table 4.** Association between demographic variables and IR

Variables	Insulin sensitive (59)	Insulin resistance (24)	P-value
**Age, years**	53.95±9.04	54.71±9.88	0.737
**Gender**			
**Male**	41 (69.49%)	12 (50%)	0.094
**Female**	18 (30.51%)	12 (50%)	
**Smoking**			
**Never**	37 (62.71%)	13 (54.17%)	0.471
**Ex/current**	22 (37.29%)	11 (45.83%)	
**Family history**			
**No**	28 (47.45%)	11 (45.83%)	0.893
**Yes**	31 (52.54%)	13 (54.17%)	
**Weight, kg**	80.92±11.84	87.21±12.12	0.029
**Height, cm**	166.17±6.02	167.92±5.25	0.218
**WC, cm**	93.17±19.37	94.04±19.17	0.852
**HC, cm**	105.64±21.5	106.0±22.34	0.946
**BMI, kg/m^2^**	28.95±3.45	31.37±3.84	0.016

WC – waist circumference; HC – hip circumference.

The mean LDL in the IR group was significantly higher (106.36±22.67 mg/dl) than that of the insulin-sensitive group (33.81±9.02 mg/dl). However, most lipid profile components were comparable between the two groups with no significant differences. The mean TG/HDL ratio in the IR group was significantly higher (3.69±0.68) than that of the insulin-sensitive group (2.71±1.0). Similarly, the mean LDL/HDL ratio in the IR group was significantly higher (2.35±0.53) than that of the insulin-sensitive group (1.97±0.4), as shown in Table 5.

**Table 5.** Association between lipid profile and IR

Variables	Insulin sensitive (59)	Insulin resistance (24)	P-value
**Total cholesterol, mg/dl**	179.86±24.35	189.96±30.45	0.116
**Triglycerides, mg/dl**	142.54±49.67	166.75±49.68	0.141
**HDL, mg/dl**	50.38±4.69	45.15±4.55	0.177
**LDL, mg/dl**	96.62±17.14	106.36±22.67	*0.036
**VLDL, mg/dl**	33.81±9.02	35.44±9.59	0.466
**TG/HDL ratio**	2.71±1.0	3.69±0.68	*0.009
**LDL/HDL ratio**	1.97±0.4	2.35±0.53	*0.019

Fasting blood sugar and fasting insulin, as components of the HOMA-IR equation, were much higher in the IR group than in the insulin-sensitive group, with highly significant differences (Table 6).

**Table 6.** Association of FBS and HbA1c with IR

Variables	Insulin sensitive (59)	Insulin resistance (24)	P-value
**FBS, mg/dl**	87.98±8.03	93.04±6.82	0.008
**HbA1c, %**	5.03±0.51	5.15±0.6	0.381
**Fasting insulin, mIU/L**	8.32±2.72	17.25±4.1	<0.001
**HOMA-IR**	1.73±0.5	3.94±0.85	<0.001

### Predictive value of TG/HDL and LDL/HDL ratio in detecting IR

**Figure 2.**
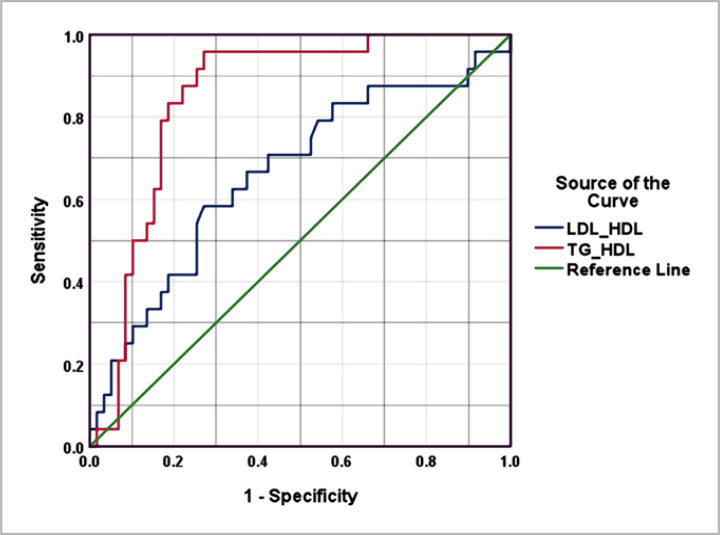
Receiver operating curve for TG/HDL and LDL/HDL ratios in detecting IR in healthy subjects.

The diagnostic value of the TG/HDL ratio in predicting IR was evaluated using receiver operating characteristic (ROC) curves. The AUC for TG/HDL ratio was 0.849, 95%CI= 0.763-0.935, p<0.002, indicating a high level of accuracy in predicting IR. The test had a sensitivity of 83% and specificity of 81%, with a best cut-off value of 3.1. Conversely, for LDL/HDL ratio, the AUC was 0.662, 95%CI= 0.528-0.707, with a sensitivity of 71% and specificity of 58%. The best cut-off value of the LDL/HDL ratio was 2.0 (Figure 2). The positive likelihood ratio (LR) was 1.48, while the negative LR was 0.5.

## DISCUSSION

The prevalence of IR among healthy adult Iraqis in this study was 28.92%. However, comparing the reported rates of IR around the world is challenging due to variations in threshold values for insulinemia and HOMA, as well as the unique characteristics of each study sample. Various authors have reported different threshold values for insulinemia and HOMA as indicators of the onset of insulin resistance (IR). Insulinemia levels typically range from 16-16.7 mU/L, while HOMA threshold values often fall between 2.0 and 4.6. A cut-off value of 2.6 for IR was adapted in the present study [9]. Worldwide IR prevalence rates range from 15.5% to 46.5% [10-12]. Compared to other studies, the present rate falls within an international context. Several studies reported higher rates of IR than our study. For example, in a cross-sectional study of 2026 adults in Venezuela, Bermudez et al. [10] found an IR rate of 46.5% using the HOMA2-IR ≥ 2 formula. A meta-analysis of 12 studies with a total of 2198 subjects by Goh et al. [13] reported that the general rate of IR in Southeast Asia was 44.3%, with the highest rates in Malaysia (50.4%) and Indonesia (44.2%). In Iran, the prevalence of IR was reported as 51% [14], while in Hispanic subjects living in Texas, USA, it was estimated to be 39.1%. In Qatar, IR prevalence in healthy, non-obese females varied from 7% to 37% according to BMI and other demographic characteristics [15]. On the other hand, several studies reported lower rates of IR than the present study. For instance, the estimated rate was 17% in a Danish study [11], and Kim et al. [16] found an incidence rate of 6.9 per 100 persons/year in the Korean population. Rogero Blanco et al. [9] also investigated IR in 118 non-diabetic young adults aged 18 and 19 years, using a cut-off value of 3.5 for IR. The study revealed a 9.3% rate of IR in individuals with healthy weight, while this figure rose to 50% in the obesity group.

The determination of IR is influenced by many social, genetic, epigenetic, and anthropometric variables [12], which can explain the variations in the rates reported in different studies. IR is a significant predictor of the risk of diabetes in non-obese people, with up to an 80% increased risk reported in previous studies [17]. Since IR typically precedes the development of T2DM by 10-15 years [18], it is estimated that approximately 25% of healthy Iraqi adults may develop T2DM within the next 15 years. In this study, body weight and BMI were significantly associated with IR, while age, smoking, family history of T2DM, height, WC, and HC had no significant association. Interestingly, the IR rate was more common among females than males, although there was no significant association. These findings are consistent with previous studies worldwide, showing that increasing age and being overweight or obese are independent risk factors for IR [19]. Additionally, Kim et al. [16] found a significant association between IR and age (relative risk (RR) = 1.03, 95% CI: 1.00-1.05) and waist-hip ratio (RR=1.74, 95% CI: 1.17-2.58) in the Korean, while Bermudez et al. [10] reported that IR rate increases with age (significantly greater in individuals ≥30 years), employment status, alcohol consumption, and obesity.

Similarly, the reduction of lean mass and the increase in visceral adiposity associated with aging have been strongly connected with IR [20]. Furthermore, there is a notable discrepancy among studies in population characteristics and IR assessment methods, but sample size can also have a significant impact. In the present study, most components of the lipid profile were not significantly associated with IR, except for LDL, which was positively associated with IR. Previous studies indicated the importance of TG and HDL as risk factors for IR. For example, Chiang et al. [21] found that TG and HDL were significantly different between individuals with and without IR, while TC and LDL showed no significant association. Similarly, a study in China with 1608 adult participants reported similar results [22]. Because insulin has an impact on how vLDL-C and HDL-C are metabolized, patients with IR frequently exhibit hypertriglyceridemia and low HDL-C levels [23]. Insulin promotes lipoprotein lipase (LPL) activity and reduces vLDL-C production. However, IR causes the liver to produce more TG, which raises apolipoprotein B levels via increasing fat generation, boosts vLDL-C secretion, and lowers LPL concentration and activity in peripheral tissues, particularly adipose tissue. Ultimately, decreased HDL-C levels are a byproduct of increased TG levels [24].

In this study, the AUC for TG/HDL in predicting IR was 0.849 (95%CI= 0.763-0.935, p<0.002). The sensitivity and specificity of the test were 83% and 81%, respectively. The best cut-off value of the TG/HDL ratio was 3.1. These findings suggest that the TG/HDL ratio could serve as an alternative surrogate to HOMA-IR in screening for IR. However, data on the effectiveness of the TG/HDL ratio in detecting IR in various populations are inconclusive, with different cut-off ratios reported in earlier research. For example, McLaughlin et al. [25] suggested a TG/HDL ratio to identify obese people who are IR, while Yang et al. [22] reported a cut-off value of 3.0 in predicting IR among overweight individuals. In a Taiwanese study, ROC analysis was conducted to evaluate the ability of TG/HDL to correctly discriminate between subjects of low and elevated HOMA-IR. The AUC was 0.71 (95% CI = 0.67-0.75) [21].

It has been documented that there are variations in TG levels and HDL-C values across different races and ethnicities. One study found that non-Hispanic blacks had lower TG concentrations compared to Mexican Americans and non-Hispanic whites [7]. To predict IR using TG/HDL-C, the researchers suggested a cut-off value of 2.0 for non-Hispanic blacks and 3.0 for Mexican Americans and non-Hispanic whites. Additionally, the study showed that individuals with a BMI under 25 kg/m2 had a greater correlation between the TG/HDL-C ratio and hyperinsulinemia [25]. However, in African Americans, it was demonstrated that TG levels and TG–HDL-c ratio are not reliable markers of IR [8]. Thus, the TG/ HDL-C ratio may be a good marker to identify individuals with IR of Aboriginal, Chinese, and European descent but not African Americans [26, 27]. The non-significant differences between patients with IR and those without IR regarding waist circumference may be attributed to the type and duration of physical activities practiced by the participants, which was not assessed.

## CONCLUSION

The study found a prevalence of IR of 28.92% among healthy Iraqi adults, with body mass index, body weight, and LDL-c being significantly associated with IR. The triglyceride/HDL-c ratio showed a very good predictive value for IR with an AUC of 0.849, 95%CI= 0.763-0.935. The sensitivity and specificity of the test were 83% and 81%, respectively. The best threshold value of the TG/HDL ratio was 3.1.
